# Continuous Fluorescent Sirtuin Activity Assay Based on Fatty Acylated Lysines

**DOI:** 10.3390/ijms24087416

**Published:** 2023-04-18

**Authors:** Matthes Zessin, Marat Meleshin, Sebastian Hilscher, Cordelia Schiene-Fischer, Cyril Barinka, Manfred Jung, Mike Schutkowski

**Affiliations:** 1 Department of Medicinal Chemistry, Institute of Pharmacy, Martin-Luther-University Halle-Wittenberg, 06120 Halle, Germany; 2 Department of Enzymology, Charles Tanford Protein Center, Institute of Biochemistry and Biotechnology, Martin-Luther-University Halle-Wittenberg, 06120 Halle, Germany; 3 Institute of Biotechnology, Czech Academy of Sciences, BIOCEV, Prumyslova 595, 25250 Vestec, Czech Republic; 4 Institute of Pharmaceutical Sciences, University of Freiburg, Albertstraße 25, 79104 Freiburg, Germany

**Keywords:** histone deacetylases, sirtuins, fluorescence quenching, sirtuin inhibitors, myristoylated substrates, continuous activity assay, bovine serum albumin effect

## Abstract

Lysine deacetylases, like histone deacetylases (HDACs) and sirtuins (SIRTs), are involved in many regulatory processes such as control of metabolic pathways, DNA repair, and stress responses. Besides robust deacetylase activity, sirtuin isoforms SIRT2 and SIRT3 also show demyristoylase activity. Interestingly, most of the inhibitors described so far for SIRT2 are not active if myristoylated substrates are used. Activity assays with myristoylated substrates are either complex because of coupling to enzymatic reactions or time-consuming because of discontinuous assay formats. Here we describe sirtuin substrates enabling direct recording of fluorescence changes in a continuous format. Fluorescence of the fatty acylated substrate is different when compared to the deacylated peptide product. Additionally, the dynamic range of the assay could be improved by the addition of bovine serum albumin, which binds the fatty acylated substrate and quenches its fluorescence. The main advantage of the developed activity assay is the native myristoyl residue at the lysine side chain avoiding artifacts resulting from the modified fatty acyl residues used so far for direct fluorescence-based assays. Due to the extraordinary kinetic constants of the new substrates (K_M_ values in the low nM range, specificity constants between 175,000 and 697,000 M^−1^s^−1^) it was possible to reliably determine the IC_50_ and K_i_ values for different inhibitors in the presence of only 50 pM of SIRT2 using different microtiter plate formats.

## 1. Introduction

Acylation of lysine side chains in proteins is a widespread posttranslational modification that is modulated by the enzymatic activity of acetyltransferases and enzymes known as histone deac(et)ylases (HDACs). HDACs are divided into 4 classes based on their sequence homology. Class I (HDAC1, 2, 3, and 8), class IIa (HDAC4, 5, 7 and 9), class IIb (HDAC6 and 10), and class IV (HDAC11) are grouped within the Zn^2+^ dependent hydrolases. Class III enzymes (called sirtuins; human SIRT1–SIRT7) require NAD^+^ as a co-substrate to transfer an acyl moiety from the lysine side chain to the ADP-ribosyl fragment of the co-substrate generating 2-O-acetyl-ADP-ribose and nicotinamide as the second and third reaction product, respectively [1].

HDACs are involved in a number of patho(physiological) processes including cancer progression, obesity, and immune function. Consequently, several HDAC inhibitors (HDACi) have already been approved by the Food and Drug Administration for the treatment of cancer (including vorinostat, romidepsin, belinostat, and panobinostat) and a number of clinical trials with sirtuin inhibitors (either natural products or synthetic small molecules) are ongoing. Robust and continuous sirtuin/HDAC activity assays compatible with high-throughput screening (HTS) are, however, required for further drug development.

Several enzymatic assays have been developed to monitor the lysine deacylase activity of HDACs/SIRTs as reviewed in [2,3]. Most of these activity assays are discontinuous (HPLC-based or mass spectrometry-based assays), or suffer from complexity due to coupled enzymatic [4,5,6,7,8,9,10,11] or chemical reactions [12] (reviewed in [13,14]).

Activity assays for lysine deacylase are often linked to a separation step enabling quantification of the peptide substrate and a reaction product. The following separation methods were used: capillary electrophoresis [15], microchip electrophoresis [16], microfluidic mobility [17,18], polyacrylamide gel electrophoresis [19], high- performance liquid chromatography [20,21,22,23], thin layer chromatography [24], charcoal binding [25], binding to boronic acid resins [26], and extraction with organic solvents [27]. However, these activity assays are discontinuous and time consuming. Mass spectrometry could be used for quantification of substrates and products subsequent to separation in the gas phase [28,29,30,31]. Alternatively, biological reagents and chemical reactions could be used for detection of acetylated substrates, such as acetyllysine-recognizing antibodies [32,33,34,35,36,37], or reaction products, such as the released primary amino function of the lysine side chain. Described are acylations with biotin-containing compounds or fluorescent dyes [38], alkylations with fluorescamine [39], or intra-molecular reactions, such as transesterification with a coumarin dye [40,41,42] or release of bioluminescent luciferin subsequent to an intra-molecular cleavage of an ester bond [43] or intramolecular aldimine formation [44,45].

**Figure 1:**
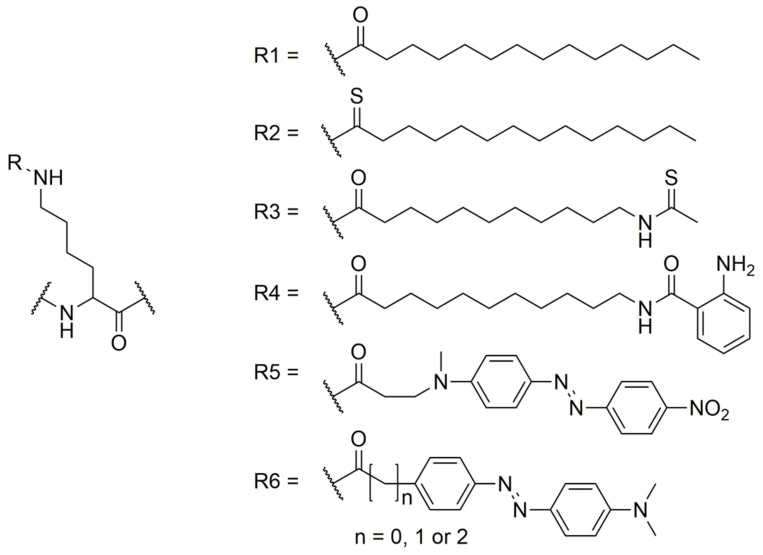
Structure of acyl residues used for different deacylase activity assays.

To develop a more robust activity assay that is suitable for the different lysine deacylase isoforms, it is important to consider the influence of the acyl residue on substrate specificity. For example, nearly all sirtuins have demyristoylation activity [46], and it has been shown that HDAC11 also has a high demyristoylase activity [47,48,49]. We recently demonstrated that the myristoylated lysine residue (Figure 1, R1) in TNFα-derived substrate peptides can be replaced by a 2-aminobenzoylated-11-amino-undecanoylated lysine residue (Figure 1, R4), generating a substrate derivative that enables continuous activity measurement of both sirtuins and HDAC11 [50,51].

It has also been shown that an increase in the size of the fluorophore, which allows fluorescence measurements at longer wavelengths, leads to decreased substrate specificity and decreased kinetic properties for sirtuins [50] and HDAC11 [51]. Because sirtuins can accept relatively large fluorophores within the peptide sequence, we addressed this issue by designing substrates where the fluorophore and quencher positions are switched [3]. Replacing the 2-aminobenzoyl residue by a 2-amino-5-nitro-benzoyl quencher resulted in SIRT2 substrates with very similar specificity constants [50]. In the search for smaller quenchers, we exploited the ability thioamides to quench fluorescence by photoinduced electron transfer (PET) [52]. We showed that replacement of the amide scissile bond in fluorescently labeled HDAC substrates by a thioamide bond enabled continuous activity determination of HDAC8 and HDAC11 [53]. Unfortunately, thioamide bonds were poor targets for sirtuins because of the generation of a so-called stalled intermediate [54], and thiomyristoyl residues (Figure 1, R2) are components of efficient inhibitors of SIRT1-3 and SIRT6 [55,56,57]. To overcome this problem, we introduced a thioamide bond into the myristoylated lysine residue (Figure 1, R3) and were able to generate substrates for sirtuins and HDAC11 that showed superior catalytic constants in a continuous and direct activity assay [3].

Additionally, Kawaguchi et al. showed that SIRT1-3 and SIRT6 are able to recognize the sterically more demanding DABCYL moiety in the acyl chain (Figure 1, R5), enabling monitoring of sirtuin activity in living cells [58]. New fluorescence probes for sirtuin activity measurements were generated by replacing the DABCYL quencher with disperse red dye (Figure 1, R6) [59]. Unfortunately, all of the continuous activity assays described suffer from the need for modified acyl moieties on the lysine side chain which can cause assay artifacts [60]. Therefore, to overcome this limitation, we searched for alternative methods to change the fluorescence intensity of a peptide substrate when compared to the corresponding deacylated product. Here, we describe the use of myristoylated peptides, representing the naturally occurring acyl modification, in combination with environmentally sensitive fluorophores for continuous and sensitive detection of sirtuin activity. Furthermore, we show that the use of bovine serum albumin (BSA) as a fluorescence quencher for the substrate dramatically enhances the dynamic range of the defatty acylase activity assay.

## 2. Results

**Figure 2:**
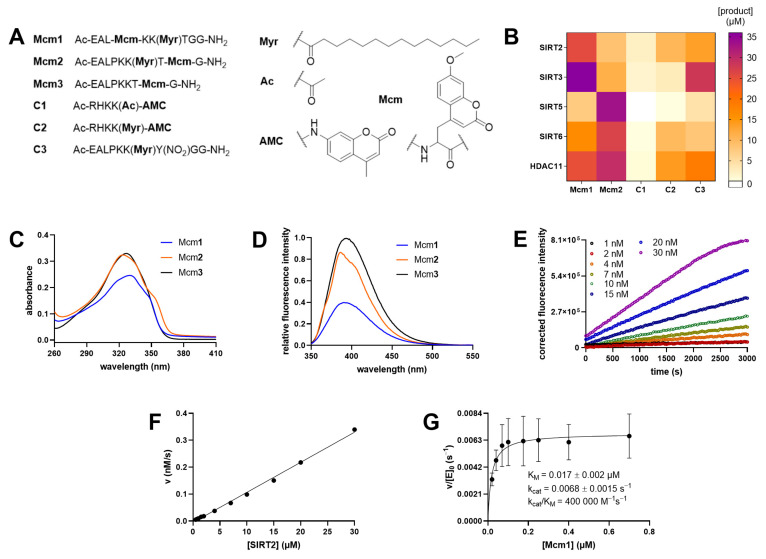
Direct and continuous activity assay for demyristoylation using the environmentally sensitive fluorophore Mcm (7-Methoxycoumarin-4-yl-alanyl). (**A**) Structures of compounds **Mcm1** to **Mcm3** and control peptides **C1** to **C3**. Y(NO_2_) corresponded to *meta*-nitrotyrosine. (**B**) Peptide substrates at an initial concentration of 50 µM were treated with either 50 nM HDAC11, or with SIRT2 (0.1 µM), SIRT3 (0.1 µM), SIRT5 (0.5 µM), or SIRT6 (0.5 µM) in the presence of 500 µM NAD^+^ at 37 °C for 60 min. Product formation was monitored via HPLC at 320 nm or 360 nm for **C3**. Values were obtained from three independent replicates. (**C**) Absorbance spectra for **Mcm1** to **Mcm3** at 30 µM concentration. (**D**) Normalized fluorescence spectra of **Mcm1** to **Mcm3** at 3 µM concentration. (**E**) Progress curves of **Mcm1** deacylation (1 µM) by SIRT2 (1nM-30nM) at 25 °C (λ_Ex_ = 330 ± 75 nm and λ_Em_ = 405 ± 8 nm) monitored in a 96-well plate format. (**F**) The reaction rate shows a linear correlation with the SIRT2 concentration. (**G**) Michaelis-Menten kinetic analysis of **Mcm1** deacylation by SIRT2 obtained from three independent replicates.

To test whether the sirtuin/HDAC-mediated release of the hydrophobic myristoyl residue from a peptide substrate could be sensed by fluorophores, we synthesized a TNFα-derived peptide [3,50] that comprised a myristoylated lysine residue in combination with a coumaryl based amino acid at the position −2 (Figure 2, **Mcm1**) or +2 (Figure 2, **Mcm2, Table S1**). In a systematic fluorophore scan, these two positions were found to be less sensitive to substitution with sterically demanding moieties [3]. Additionally, we synthesized the corresponding fluorescently labeled peptide product of the SIRT/HDAC11-mediated deacylation of **Mcm2** (**Mcm3**, **Figure 2 and Figure S1–S3**). First, using an HPLC-based activity assay, we analyzed how efficiently **Mcm1** and **Mcm2** were deacylated by different SIRTs and HDAC11 (Figure 2B). Both compounds showed good substrate properties for all the tested sirtuins (including SIRT5) and for HDAC11. Second, we recorded absorbance and fluorescence spectra for **Mcm1-3** (Figure 2C,D), and differences in the latter enabled us to monitor the time-dependent demyristoylation of **Mcm1** by SIRT2 (Figure 2E), which was proportional to the enzyme concentration (Figure 2F). Non-linear analysis of the dependence of the demyristoylation rate on the substrate concentration allowed us to calculate K_M_ and k_cat_ values (Figure 2G). The K_M_ value of 17 nM was relatively low, but was in accordance with the low K_M_ value determined for the structurally related, TNFα-based substrate with the thioacetylated 11-amino undecanoic acyl residue (Figure 1, R3) [3].

**Figure 3:**
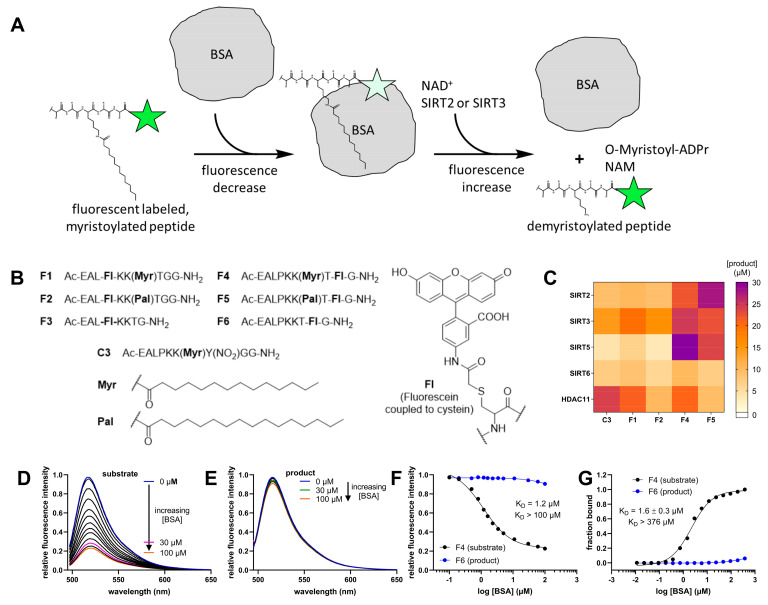
BSA-based activity assay. (**A**) The principle of the BSA-based assay. A fluorescently labeled and myristoylated peptide binds to BSA and the fluorescence is quenched. SIRT2 separates the myristoyl residue from the fluorescently labeled peptide product, which is not able to bind to BSA so tightly. (**B**) The structures of the peptides used for these experiments. Fl, or fluorescein corresponds to 5-acetamidofluorescein coupled to a cysteine residue. (**C**) Product formation of different peptide substrates at a concentration of 50 µM with subsequent treatment for 1h at 37 °C with 50 nM HDAC11, or with SIRT2 (0.1 µM), SIRT3 (0.1 µM), SIRT5 (0.5 µM) or SIRT6 (0.5 µM) in the presence of 500 µM NAD^+^. Product formation was monitored via HPLC at 320 nm (or 360 nm for **C3**). Values were obtained from three independent replicates. (**D**) Fluorescence spectra of **F4** with increasing concentrations of BSA resulting in a decrease in the fluorescence intensity of the substrate. The excitation wavelength was 490 nm. (**E**) Similar experiment to that in D, with the product **F6** showing only a slight decrease in fluorescence. The excitation wavelength was 490 nm. (**F**) Plotting the relative fluorescence intensities from **F4** and **F6** directly from (**D**,**E**) at λ_Em_ = 517 nm against the concentration of BSA yielded a K_D_ value of 1.2 µM for the **F4**/BSA complex. The K_D_ value was obtained from one experiment (*n* = 1). (**G**) Determination of the dissociation constants of **F4** and **F6** complexes with BSA measured via fluorescence polarization. The K_D_ value was obtained from three independent replicates.

The suboptimal spectral properties of **Mcm1** and **Mcm2** prompted us to evaluate substrates where Mcm residues are replaced with fluorescein covalently bound to the side chain of cysteine (Figure 3B). A similar structural element has already been described in combination with acetylated peptides derived from histone H4 to generate fluorescent reporters for monitoring histone acetyltransferase activities [61]. Additionally, we have recently shown that SIRT2 accepts fluorescein-labeled cysteine residue substrates [3]. We synthesized substrates **F1**, **F2**, **F4**, and **F5** that differed in the position of the fluorophore (−2 or +2 position) and the nature of the fatty-acyl residue (either myristoyl (**F1** and **F4**) or palmitoyl (**F2** and **F5**)) (Figure 3B and Figure S4–S10). Additionally, we synthesized the expected fluorescently labeled peptide reaction products **F3** (fluorophore in −2 position) and **F6** (fluorophore in +2 position) (Figure 3B).

As a control substrate, we synthesized a myristoylated, TNFα-derived peptide without a fluorophore but with a *meta*-nitrotyrosine residue in the +1 position, allowing more convenient detection in HPLC-based activity assays (**C3** in Figure 3B) [50]. All peptides were then evaluated as putative substrates for sirtuins 2, 3, 5, and 6, and HDAC11 and the results are shown in Figure 3C. Peptides **F4** and **F5,** both containing the fluorophore in the +2 position, turned out to be very good SIRT2 substrates. Additionally, **F4** proved to be a surprisingly good substrate for SIRT5, which is known to be specific for negatively charged acyl residues such as malonyl [62], succinyl [23], and glutaryl [63] residues. Nevertheless, the enzyme concentration for SIRT5 used was five-fold higher when compared to SIRT2 or SIRT3.

In order to develop a continuous fluorescence-based activity assay for SIRTs/HDAC11 using substrates **F1**, **F2**, **F4**, and **F5**, we compared their fluorescence spectra with the spectra of their respective deacylation products **F3** and **F6** in a protein-free assay buffer, and surprisingly we did not observe any significant differences. However, marked differences in the fluorescence spectra of substrate/product pairs in a buffer containing bovine-serum albumin (BSA), a typical component of our HDAC assay buffers, were noted. This observation suggested interactions between the substrates and BSA that were likely mediated by binding sites for fatty acids on the BSA surface [64]. 

Figure 3A shows the reaction principle behind the idea to use BSA as a discriminator between the fluorescently labeled substrate and the peptide product of the deacylase-mediated reaction. Here, a fluorescently labeled and myristoylated peptide binds tightly to BSA [64], and its fluorescence is quenched. Following deacylation by SIRTs, the reaction product is “released” from BSA, leading to a marked increase in fluorescence. Therefore, we first determined the dissociation constant of the **F4**/BSA complex using either fluorescence spectroscopy (Figure 3F) or fluorescence polarization measurements (Figure 3G). The K_D_ values were 1.2 µM and 1.6 µM, respectively. At the same time, the calculated dissociation constant for the peptide reaction product **F6** was more than 100-fold higher in both cases.

**Figure 4:**
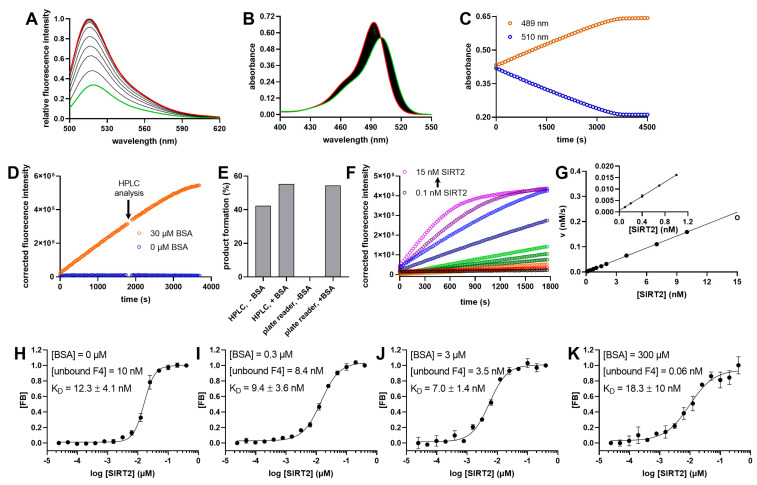
Continuous activity assay for SIRT2 using **F4** as a substrate. (**A**) Fluorescence spectra of 1µM **F4** subsequent to treatment with 30 nM SIRT2 in the presence of 500 µM NAD^+^ and 30 µM BSA. The green line shows the first, and the red line the last, spectrum recorded (total recording time 30 min). (**B**) Absorbance spectra of 10 µM of **F4** after the addition of 100 nM of SIRT2 in the presence of 500 µM of NAD^+^. The green line shows the first, and the red line the last, spectrum recorded (total recording time 30 min). (**C**) Progress curves at two different wavelengths extracted from (**B**). (**D**) Progress curves of SIRT2-mediated cleavage of **F4** measured in a 96-well plate format as changes in fluorescence intensities. After 1800 s, the measurement was interrupted, and the reaction mixture from individual wells was analyzed by HPLC. The fluorescence detection was continued afterwards. (**E**) Determination of the product formation of the reaction solution of (**D**) using HPLC with an absorbance detection at 450 nm and the fluorescence intensity readout of a 96-well MTP reader. Product formation in the MTP experiment was calculated using the total change in the fluorescence intensity as 100%. (**F**) Cleavage of 0.25 µM of **F4** mediated by different concentrations of SIRT2 in the presence of 500 µM of NAD^+^ at room temperature. Detection was performed via changes in fluorescence intensity with λ_Ex_ = 485 ± 14 nm and λ_Em_ = 535 ± 25 nm. All fluorescence values were corrected with a negative control without enzymes. The rate of this reaction was plotted as a function of the SIRT2 concentration in (**G**), and shows a linear relationship up to a SIRT2 concentration of 10 nM. (**H**–**K**) Binding curves of F4 (10 nM) to SIRT2 in the absence of NAD^+^ at different concentrations of BSA, monitored via fluorescence polarization and with three independent replicates. FB signifies the fraction bound to SIRT2.

This remarkable discrimination could be visualized by recording fluorescence spectra of **F4** and **F6** in the presence of different BSA concentrations (Figure 3D and Figure 3E, respectively) and demonstrates a strong fluorescence quenching for **F4** bound to BSA. Because of its high K_D_ value, fluorescence quenching of **F6** caused by binding to BSA was negligible within the concentration range used. Thus, deacylase-mediated removal of the fatty-acyl residue from the lysine side chain resulted in a fluorophore-labeled peptide product that no longer bound to BSA, yielding a robust increase in fluorescence over time (Figure 4A).

Deacylation of the substrate **F4** could also be monitored by measuring differences in the absorbance spectra (Figure 4B) either at 510 nm (signal decrease) or at 489 nm (signal increase) (Figure 4C). Figure 4D shows the progress curves for **F4** deacylation by SIRT2 in the assay buffer in the absence or presence of 30 µM of BSA using a 96-well microtiter plate (MTP) format, which was corrected using a control assay using the same buffer but without SIRT2. Almost no change in fluorescence intensity was observed without BSA in the reaction solution. In contrast, a strong increase in fluorescence over time in the presence of BSA could be detected. We analyzed samples of the reaction solutions with HPLC using both UV-Vis and fluorescence readouts and compared them with the results from the MTP-based format. Our data show that the fluorescence intensity in the presence of BSA in the MTP-based experiment correlated well with the product formation calculated from the HPLC-based experiments (Figure 4E). In contrast, there was no correlation between the MTP-fluorescence and HPLC readouts in the absence of BSA. This discrepancy could be explained by the effect of BSA shown in Figure 3A. SIRT2 was able to cleave the substrate in the absence of BSA, as shown by HPLC measurements, but despite the virtually identical fluorescence intensities of the substrate/product pair, no increase in fluorescence signal could be detected over time in the MTP format.

These positive results prompted us to follow the SIRT2-mediated cleavage of **F4** over a range of enzyme concentrations between 100 pM and 20 nM (Figure 4F,G) using the fluorescence readout (Figure 4F,G). The signal intensity showed a linear correlation with the SIRT2 concentration within the range of 100 pM to 10 nM. To our knowledge, this represents one of the most sensitive SIRT2 activity assays described so far. 

In the assay solution, SIRT2 and BSA compete for binding to the fluorescently labeled substrate. Therefore, we next analyzed the binding of the substrate **F4** to SIRT2 in the absence of NAD^+^ but in the presence of different BSA concentrations using fluorescence polarization measurements (Figure 4H–K). The aim of this experiment was to elucidate any impact of BSA concentration on the K_D_ value of the SIRT2/**F4** interaction. The calculated K_D_ value for binding of **F4** to the active site of SIRT2 was between 7 nM and 18 nM for BSA concentrations varying from zero to 300 µM. Thus, binding to BSA did not influence the formation of the Michaelis complex between SIRT2 and **F4**. Encouraged by these results, we determined the kinetic constants of the novel substrates for SIRT2 and SIRT3 (Table 1). SIRT5, SIRT6, and HDAC11 were not functional in this assay format because of the suboptimal affinities to the substrates **F1**, **F2**, **F4,** and **F5**.

**Figure 5:**
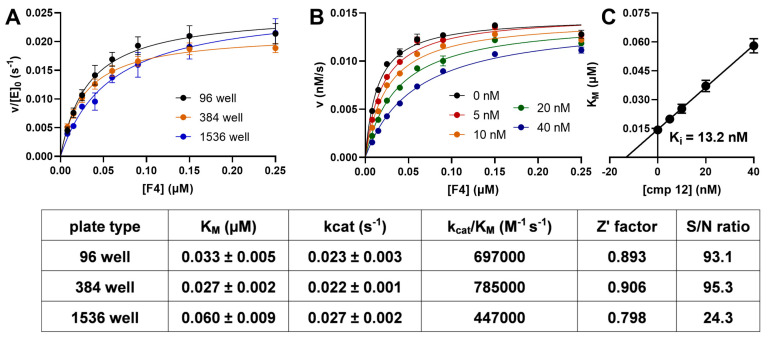
v/[S] plots of SIRT2 with F4 under different conditions. (**A**) The kinetic parameters for SIRT2 and **F4** in a 96-well MTP format compared to 384- and 1536-well MTP formats. The resulting kinetic parameters and the Z′-factors together with the respective S/N ratios for a 90% processed reaction mixture with SIRT2 and **F4** at a 1 µM concentration are summarized in the table under the figure. Data points used for the Z′-factor calculation can be found in Figure S11. The values in the table represent the mean of three independent replicates. (**B**) v/[S] plot of SIRT2 (1 nM) with different concentrations of **F4**, 500 µM of NAD^+^, and different concentrations of **cmp12** [66]. A nonlinear regression analysis was done according to the Michaelis-Menten equation. Lineweaver-Burk and Hanes-Woolf plots for these data can be found in Figure S14. (**C**) Plot of the K_M_ values from panel B as a function of the inhibitor concentration. The intercept with the abscissa represents the negative K_i_ value.

Two major conclusions can be drawn from these data. First, palmitoyl residues at the lysine side chain were accepted by SIRT2 and SIRT3, but with slightly increased K_M_ values, resulting in an approximately 2-3-fold decreased in specificity constants. Acceptance of a palmitoylated lysine residue by SIRT2 has been reported, but was not compared to myristoylated lysines [65]. Second, SIRT3 seemed to be sensitive to the position of the fluorophore in the substrate sequence. While SIRT2 did not distinguish between the −2 and +2 positions, SIRT3 accepted the bulky fluorophore in the +2 position only (Table 1). Determination of the kinetic constants for **F4** and SIRT2 using different MTP formats (Figure 5A) revealed a good correlation with excellent Z′-factors and signal to noise (S/N) ratios between 25 (1536-well MTPs) and higher than 90 (96-well and 384-well MTPs) (Figure 5).

Several known SIRT2 inhibitors (e.g., **AGK2** and **SirReal2** [67]) are very potent when acetylated substrates are used, but are much less effective with myristoylated substrates [68]. It should therefore be feasible to use the assay developed here to identify inhibitors of sirtuin-mediated defatty acylation in a continuous and direct format using naturally occurring acyl chains in the substrates.

Recently, novel derivatives of SirReal have been described (i.e., **cmp12**; the structure is shown in Figure S13) which potently inhibit SIRT2-mediated demyristoylation reactions in vitro and in cells [66]. We determined the respective K_i_ value for **cmp12** using the substrate **F4** and SIRT2. Figure 5B shows the v/[S]-plots for different concentrations of the inhibitor, indicating competitive inhibition of SIRT2 when the myristoylated peptide substrate was used. This finding is in line with the proposed binding mode of the inhibitor. Additionally, the calculated K_i_ value of 13 nM (Figure 5C) for **cmp12** was lower than any other K_i_ value reported for small molecule SIRT2 inhibitors [66]. In order to validate the substrate **F4**, we compared IC_50_ values of known SIRT2 inhibitors (including **AGK2** [69], **KK-22** [70], **S2iL5** [71], and **SMyr** [55]) using different substrates described in continuous and discontinuous activity assays and with both acetylated and myristoylated lysine residues (Table 2). The substrate **S1** represents a peptide derivative with an aminobenzoylated 11-aminoundecanoylated lysine side chain (Figure 1, R4, and Figure S11) described in [50]. The substrate **S2** represents a fluorescently labeled peptide with a thioacetylated 11-aminoundecanoylated lysine side chain (Figure 1, R3, and Figure S11) as a PET quencher described in [3]. In order to correct for the nanomolar K_M_ values of the substrates **F4** and **S1** [3], we performed these measurements either at 1 µM or at concentrations close to the K_M_ value of the respective substrate. Substrates **C1** and **C2** (Figure 2A) with acetylated and myristoylated lysines, respectively, were included because such fluorescently labeled derivatives are often used in a discontinuous assay format with trypsin as a developer protease. As seen in Table 2, inhibition potency for compounds thought to bind in the vicinity of the lysine channel (i.e., **KK-22**, **SMyr**, **S2iL5**, and **cmp12**) increased with decreasing concentration of myristoylated substrates **F4** or **S2**. In contrast, NAM inhibition showed the opposite effect, specifically for substrates **F4** and **S2** (Table 2).

Interestingly, we detected some weak inhibition for **SirReal2** using substrates **S1** (IC_50_ of 5.5 µM) and **S2** (IC_50_ = 1µM), which both contained a hydrophobic acyl residue which was structurally related to the myristoylated lysine (Figure S12). Clearly, substrates **S1** and **S2** were more sensitive to inhibition, similar to acetylated substrates. When **SirReal2** was analyzed with histone H3-derived substrates bearing a myristoylated lysine side chain at position 9 using an HPLC-based activity assay, no inhibition could be detected (IC_50_ ≥ 100 µM) [68]. We analyzed **SirReal2** inhibition using a 100 nM substrate concentration, which was most probably higher than the expected K_M_ value. Using either **F4** or **Mcm1** at concentrations above their respective K_M_ constants failed to reveal any inhibition of SIRT2 by **SirReal2** (Table 2, data lines 1, 5, and 6). When we lowered the substrate concentration of **F4** to close to the K_M_ value, the estimated IC_50_ value for **SirReal2** was close to 20 µM (Table 2, data line 2). These data demonstrate that, in contrast to the substrates **S1** and **S2**, our novel substrates **F4** and **Mcm1** behaved comparably with the themyristoylated peptides used in HPLC-based discontinuous assays, and could be used to determine the inhibition potency of compounds for SIRT2-catalyzed demyristoylation reactions.

## 3. Discussion

It was demonstrated that enzymes could be used to monitor changes in the substrates or products of sirtuin/HDAC reactions. One of the first continuous sirtuin assays was developed using a combination of nicotinamidase and glutamate dehydrogenase for indirect spectrophotometric determination of sirtuin-mediated release of nicotinamide [11]. In a similar way, a cascade of enzymatic reactions was used to quantify the remaining sirtuin cosubstrate NAD^+^ [17]. These two assay principles were shown to be independent of the chemical nature of the acyl residue, thereby allowing activity measurements with the native acyl chain, such as myristoylated substrates. Nevertheless, the combination of several enzymatic reactions with the sirtuin/HDAC reaction resulted in a complex assay setup with a limited linear range which made the assay more susceptible to artifacts by additional modulation of the enzymatic activity of the helper enzymes. This effect was demonstrated for the Sirt5 inhibitor GW5074, which affected the enzymatic activity of the helper enzyme glutamate dehydrogenase [72]. Alternatively, the deacylated peptide product of the sirtuin reaction could be quantified by coupling to the action of a protease specific for free lysine [22,73,74,75]. A fluorophore in the +1 position of the sirtuin substrate is necessary for such reagents to generate bright fluorescence subsequent to protease-mediated cleavage of the lysyl-fluorophore amide bond [5,6,7,8,9,10]. Proteolytic instability of different HDACs and sirtuins forced a discontinuous assay format, but for some sirtuin isoforms, this assay could be performed in a continuous manner [76,77]. Nevertheless, the protease-coupled assay format allowed sirtuin activity measurements using native acyl residues at the lysine. For most of the published sirtuin assays, the substrate properties are suboptimal with regard to both K_M_ and k_cat_ values, resulting in specificity constants in the range of 10 M^−1^s^−1^–10,000 M^−1^s^−1^. These constraints result in high substrate and sirtuin concentrations. Assay protocols using enzyme concentrations in the range of 500 nM (sirtuin 2) to 2 μM for sirtuin 6 reduce the validity of the Michaelis-Menten equation and prevent the determination of IC_50_ values for inhibitors binding with affinities below 250 nM. 

In contrast to most HDACs, sirtuins are able to accept longer acyl chains at the lysine side chain [46]. Based on the observation that sirtuins are able to recognize fatty acyl chains [46], several direct and fluorescence-based activity assays have been developed [50,58,59]. One of these assays was adapted to monitor HDAC11 activity [51]. A small fluorophore (or a small quencher) was fused to the acyl moiety linked to the lysine side chain (Figure 1, R3 and R4, substrate S1). The resulting substrates had good properties for Sirt2 and HDAC11, with k_cat_/K_M_ values up to 175,000 M^−1^s^−1^ and 11,000 M^−1^s^−1^, respectively [50]. Additionally, we replaced the scissile bond with a thioamide bond and were able to show that such a modification was tolerated by HDAC isoforms such as HDAC8 and HDAC11, yielding internally fluorescence-quenched HDAC8 substrates with specificity constants up to 450,000 M^−1^s^−1^. Because a similar thioamide substitution resulted in very slow substrates for sirtuins [54], we wondered if a thioamide bond within the fatty acyl residue (Figure 1, R3) would result in a small quencher. In combination with a respective fluorophore in the peptide chain, the thioacylated 11-aminoundecanoylated lysine derivatives (Figure 1, R3) showed extremely low K_M_ values, down to 1 nM for sirtuin 2 [3]. The known K_M_ values for continuous assay substrates for sirtuin 2 are 120 nM [50], 520 nM [58], and 41 nM [59]. The data presented in Table 2 show that substrates with modified fatty acyl residues such as S1 and S2 do not match the properties of the native myristoyl residue. Determination of the IC_50_ values of SirReal2 for the sirtuin 2-catalyzed reaction showed obvious inhibition when hydrophobic substrates S1 and S2 were used, but no inhibition when **F4** or Mcm1 were utilized. Therefore, our novel substrates only reflect the influence of compounds on the demyristoylation activity of sirtuin 2 and other isoforms.

Based on the low K_M_ value of **F4**, SIRT2 activity could be reliably detected in microtiter-plate formats with enzyme concentrations as low as 100 pM and a substrate concentration of 40 nM. To the best of our knowledge, this represents one of the most sensitive SIRT2 substrates described so far, enabling highly effective inhibitor screening projects because of the reagent-saving activity assay format. Additionally, substrates **F1** and **F2** could be used for selective monitoring of sirtuin 2 activity in more complex biological fluids such as cell lysates or within cells, because in contrast to **F3** and **F4**, they could not be cleaved by SIRT3 (Table 1). Nevertheless, metabolic stability must be increased. Cleavages of peptide bonds between the fluorophore and the fatty acylated lysine by proteases yielded a false positive signal. Replacement of amino acid residues by either D-amino acids or N-methyl amino acids are known to prevent proteolytic cleavage. In a systematic study, we were able to demonstrate broad acceptance of such modifications in sirtuin substrates. For cell-based experiments, the metabolically stabilized substrate could be fused to oligo-arginines for better cell penetration. We know that sirtuins recognize peptide substrates fused via the N-terminus to oligo(deca)-D-arginine, opening the way for such substrates to be applied in living cells. Two different methods have been described for sirtuin activity measurements in living cells. Sirtuin activity could either be monitored by spontaneous chromophore maturation after deacetylation of lysine 85 in enhanced green fluorescent protein [78], or by replacing the lysine 529 in the active site by an acetylated lysine in firefly luciferase results in an enzymatically inactive enzyme variant. Sirtuin-mediated deacetylation could be monitored in a continuous assay format by restored luciferase activity [79].

In summary, we were able to demonstrate that environmentally sensitive fluorophores could be helpful for the development of efficient sirtuin substrates, which were useful for continuous activity measurements with microtiter plate-based equipment. The superior kinetic constants of substrate F4 enabled the determination of inhibition constants with substrate concentrations in the low nanomolar range, and SIRT2 concentrations down to 100 pM. Moreover, the developed substrates such as F1 and F2 represent a starting point for the generation of probes enabling monitoring of SIRT2 activity in vivo.

## 4. Materials and Methods

### 4.1. Enzymes and Chemicals

Sirtuins and HDAC11 were expressed and purified as described previously [3,47]. All chemicals were purchased from Sigma-Aldrich (St. Louis, MO, USA) unless otherwise indicated. N,N-dimethylformamid (DMF), piperidine, ethyl(hydroxyamino)cyanoacetate (OxymaPure), pentafluorophenol, and rink amide MBHA were purchased from Iris Biotech (Markredwitz, Germany). The 9-fluorenylmethoxy-carbonyl- (Fmoc)-protected amino acid derivatives and O-(benzotriazol-1-yl)-N,N,N′,N′-tetramethyluronium hexafluorophosphate (HBTU) were purchased from Merck (Darmstadt, Germany). Trifluoroacetic acid (TFA) was obtained from Roth (Karlsruhe, Germany). Fmoc-protected β-(7-methoxy-coumarin-4-yl)-alanine (Mcm) was purchased from Bachem (Bubendorf, Switzerland). **KK22**, **cmp12, S2iL5,** Fmoc-Lys(Ns)-OH, and **SMyr** were synthesized as described previously [50,66,70]. SirReal2 was purchased from Biomol (Hamburg, Germany). **AGK2** was purchased from Selleckchem (Houston, TX, USA).

For all HPLC purifications and analyses, a system of water (solvent A) and acetonitrile (solvent B), both supplemented with 0.1% (*v*/*v*) trifluoroacetic acid (TFA), was used. Purification of compounds was done using a Shimadzu LC System (Kyoto, Japan) with a Phenomenex (Torrance, CA, USA) Kinetex^TM^ 5 μm XB-C18 column (250 × 21.1 mm, 100 Å) at a flow rate of 15 mL/min. Different gradients were used depending on the compound, with a runtime of 45 min. Analytical HPLC runs were performed using an Agilent 1100 HPLC system (Santa Clara, CA, USA) with a well plate autosampler and a diode-array detector. 

UPLC-MS analysis was carried out using either a Waters Acquity UPLC-MS system or a Waters XEVOTQD UPLC-MS system (Milford, MA, USA) with a Waters Acquity-UPLC-MS-BEH C18 column of 1.7 μM (2.1 × 50 mm; 30 Å). A system of water (solvent A) and acetonitrile (solvent B) supplemented with 0.1% (*v*/*v*) formic acid was used for LC analysis, with a typical gradient from 5 to 95% of solvent B within 6 min, and a flowrate of 0.6 mL/min. Data analysis was done using the Waters software MassLynx 4.1.

### 4.2. Synthesis

The peptides were synthesized using an automated microwave peptide synthesizer, Liberty Blue^TM^ from CEM Corporation (Matthews, NC, USA). A Fmoc-based solid-phase peptide synthesis (SPPS) strategy with rink amide MBHA resin was used. The amino acid coupling was performed twice for every amino acid at 90 °C for 2 min with OxymaPure/DIC in DMF. Fmoc-deprotection was done with 20% (*v*/*v*) piperidine for 1 min at 90 °C, and the final N-terminal deprotection was done with a 1:2:7 mixture of acetic anhydride/DIPEA/DMF (*v*/*v*) for 1 h at room temperature. 

### 4.3. Mcm1–Mcm3

The Mcm fluorophore was introduced as a Fmoc-ß-(7-methoxy-coumarin-4-yl)-Ala-OH building block via SPPS, and the myristoyl modification of the ε-amino group of the lysine was introduced as a Fmoc-Lys(Myr)-OH building block.

### 4.4. F1–F6

The peptides were synthesized via SPPS, and the resin modification a 2-nitrobenzenesulfonyl (nosyl) protected lysine (Fmoc-Lys(Ns)-OH) was introduced during synthesis. The nosyl-protecting group was cleaved using a 1,8-diazabicyclo[5.4.0]undec-7-en/thiophenol/DMF solution (1.5/1/7.5 *v*/*v*) for 90 min, and the procedure was repeated a second time. **F3** and **F6** were cleaved after washing. After washing with DMF, coupling of the myristoyl residue (**F1** and **F4**) was done with 4 equivalents of Myristoyl chloride for 1h, and coupling of the palmitoyl residue (**F2** and **F5**) was done with 4 equivalents of palmitoyl chloride. After washing with DCM (4 × 5 min), methanol (2 × 5 min) and DCM (4 × 5 min) peptides were cleaved of the resin with a TFA/TIPS/H_2_O (95/2.5/2.5 *v*/*v*) solution twice for 1 h. TFA was removed in vacuo, and the residual solution was dissolved in water/acetonitrile solution (1/1 *v*/*v*) and purified via HPLC. Pure peptide-containing fractions were combined and lyophilized. A total of 5 mg of 5-iodoacetamido fluorescein was coupled with 1.2 equivalents of pure peptide and 6 equivalents of DIPEA in DMF for 1 h. The solution was injected directly into the HPLC system, and peptide-containing fractions were collected, frozen and lyophilized.

### 4.5. Recording of Fluorescence and Absorbance Spectra

All spectra were recorded in a SIRT assay buffer consisting of 20 mM of Tris-HCl, pH 7.8, 150 mM of NaCl, and 5 mg/mL of MgCl_2_. The absorbance spectra were recorded at room temperature in a cuvette with a pathlength of 10 mm using an Agilent CARY 3500 UV-Vis spectrometer. A compound concentration of 30 µM for Mcm-containing compounds or 10 µM for fluorescein-containing compounds was used. The fluorescence spectra were recorded in a fluorescence cuvette with a pathlength of 10 mm × 5 mm with a Horiba Fluoromax 4 (Kyōto, Japan). The compound concentration was 3 µM for Mcm-containing peptides and 1 µM for fluorescein-containing peptides. Excitation wavelengths were chosen according to the absorbance maximum of the individual compounds.

### 4.6. Quenching Efficiency Determination

Quenching efficiency was determined from the fluorescence emission spectra with the following equation at a wavelength of 515 nm. Fi_acyl_ is the fluorescence intensity of the fluorescence peptide with a myristoyl residue, and Fi_free_ is the fluorescence intensity of the deacylated counterpart.

$$Q_{E}=100*\left(1-\frac{{\mathrm{Fi}}_{\mathrm{acyl}}}{{\mathrm{Fi}}_{\mathrm{free}}}\right)$$

### 4.7. HPLC Based Deacylation Analysis

The HPLC analysis was done in a total volume of 70 µL of SIRT assay buffer (composition above) supplemented with 2 mg/mL BSA for sirtuins, or in HDAC11 assay buffer (20 mM HEPES, 70 µM TCEP, and 2 mg/mL BSA, pH 7.4 adjusted with NaOH) for HDAC11. The reaction mixture with 50 µM of peptide and 500 µM of NAD^+^ (only for sirtuins) was incubated for 5 min at 37 °C, and the reaction was started with the addition of an enzyme ([SIRT2] and [SIRT3] = 100 nM, [SIRT5] and [SIRT6] = 500 nM, and [HDAC11] = 50 nM final concentration). After 0.5 h and 1 h, the reaction was quenched by adding 20 µL of a stop solution (5% acetonitrile (*v*/*v*) and 1% TFA (*v*/*v*) in water), and the solution was injected directly into the HPLC-system. Separation of the substrate and product was done with a linear gradient from 5% to 95% of solvent B within 6 min and at a flow rate of 0.6 mL/min. Detection was done at a wavelength of 320 nm for **Mcm1**, **Mcm2**, and **C1** and **C2**, at 360 nm for **C3,** and at 450 nm for **F1**, **F2**, **F4**, and **F5**. Product formation was calculated as the ratio of product peak area to total peak area.

### 4.8. Steady-State Kinetics

The steady-state measurements were done using the sirtuin-mediated fluorescence change through product formation. The reaction took place in a black 96-well fluorescence plate in a total volume of 100 µL. The peptide was incubated at different concentrations (depending on the substrate properties) with 500 µM of NAD^+^ (final concentration) in SIRT assay buffer (composition above) supplemented with 2 mg/mL of BSA (0.1 % (*w*/*v*) Tween20 for **Mcm1**) for at least 5 min at 25 °C. The reaction was started by the addition of an enzyme (1 nM SIRT2 or 10 nM SIRT3 for **F4** and **F5**; 20 nM SIRT3 for **F1**; and 50 nM for **F2**), and the fluorescence change was monitored continuously with a PerkinElmer Envision 2104 multilabel plate reader (Waltham, MA, USA). The excitation wavelength was set to λ_Ex_ = 485 ± 14 nm, and the emission wavelength was set to λ_Em_ = 535 ± 25 nm for **F1** to **F6,** and to λ_Ex_ = 320 ± 14 nm and λ_Em_ = 405 ± 8 nm for **Mcm1**. The fluorescence intensities were plotted as a function of time, and the slope of the linear part of this curve represented the reaction rate (steady state velocity). The product concentration was calculated using calibration lines, the reaction rate was plotted against the substrate concentration, and a fit according to the Michaelis-Menten equation was done to determine the K_M_ and the k_cat_ values.

### 4.9. Determination of IC_50_ Values

The inhibition experiments were done using different assays depending on the substrate. For peptides **F4**, **Mcm1**, **S1**, and **S2**, the enzyme activity was determined in a continuous manner. For **C1** and **C2**, a trypsin-coupled discontinuous endpoint assay was used. For all assays, a dilution series of the inhibitor was done in DMSO.

The continuous assays were performed in a black 384-well fluorescence plate in a total volume of 40 µL of SIRT assay buffer (composition above) supplemented with 2 mg/mL of BSA for **F4**, **Mcm1**, **S2,** and **S1**, or 0.1% Tween20 for **Mcm1**. A total of 2 µL of the inhibitor dilutions were incubated with 14 µL of the peptide substrate (final concentration 1 µM for **F4**, **Mcm1**, **S1**, and **S2**, 40 nM for **F4,** or 10 nM for **S2**) and 14 µL of SIRT2 solution (final concentration 10 nM for **F4**, **Mcm1**, **S1**, and **S2,** 1 nM for **F4**, or 0.5 nM for **S2**) for 5 min at room temperature. In addition to the inhibitor-containing samples, a sample without inhibitors was measured as a positive control, and a sample without enzymes was measured as a negative control. The DMSO concentration was set to 5 % (*v*/*v*). The reaction was started with the addition of 10 µL of NAD^+^ (final concentration 500 µM), the product formation was monitored by the change of the fluorescence intensity, and the reaction rate (see above) represented the activity. Fluorescence readouts for **S2** were performed at the same wavelengths as **F4,** and readouts for **S1** were performed at the same wavelengths as **Mcm1**. The activity was normalized using the positive control as 100% and the negative control as 0%. The normalized activity (*v*/*v_0_*) was plotted as a function of the logarithm of the inhibitor concentration [I], and a nonlinear fit was performed according to the following equation to determine the IC_50_ value.

$$\frac{v}{v_{0}}=\frac{100}{1+10^{\left[I\right]-{\mathrm{logIC}}_{50}}}$$

The discontinuous fluorescence assay was performed in a black 384-well fluorescence plate in a total volume of 21 µL (for the sirtuin reaction) with **C1** (for the deacetylation reaction) and **C2** (for the demyristoylation reaction) used as peptide substrates. A total of 1.05 µL inhibitor solution was incubated with 9.95 µL SIRT2 solution (final concentration of 100 nM) for 5 min at room temperature, and the reaction was started with the addition of substrate solutions with final concentrations of [**C1**] = 20 µM or [**C2**] = 1 µM and 500 µM NAD^+^. Additionally, a positive control without inhibitor and a negative control without enzyme were performed in the same plate. After 2 h incubation at 37 °C for **C1** and 1 h for **C2**, the reaction was stopped, and the fluorescence signal was developed upon the addition of 21 µL of developer solution containing trypsin (0.5 mg/mL final concentration) and nicotinamide (1 mM final concentration) and additional incubation for 1 h. The fluorescence readout was performed with the 2104 Envision Multilabel plate reader with λ_Ex_ = 380 ± 10 nm and λ_Em_ = 475 ± 8 nm. The fluorescence signal of the positive control was set to 100%, the fluorescence signal of the negative control was set to 0%, and the other values were normalized using these two values. The normalized fluorescence intensities represent the activity. The IC_50_ calculation was done as described above.

### 4.10. Determination of the K_i_ Values

Determination of the K_i_ values was done in the same way as the IC_50_ determination, but with different substrate concentrations and in a black 96-well plate. The total volume was 100 µL per well. The inhibitor was incubated with peptide substrate **S2** or **F4** and 0.5 nM or 1 nM SIRT2 for 5 min at room temperature, and the reaction was started with the addition of NAD^+^. The progress of the reaction was monitored via the fluorescence intensity, as described above. The reciprocal reaction rate was plotted as a function of the reciprocal substrate concentration for each inhibitor concentration to determine the inhibition type. For the competitive inhibitor **cmp12**, the slope of these plots at different inhibitor concentrations was plotted against the inhibitor concentration, and the linearity of these plots determined the inhibition type. The intercept with the abscissa represents the negative K_i_ value.

### 4.11. Binding Experiments

All binding experiments were performed in a black 384-well plate with a reduced volume (total volume of 20 µL). The experiment was done in SIRT assay buffer, containing 20 mM of Tris-HCl, pH 7.8, 150 mM of NaCl, and 5 mM of MgCl_2_. The unlabeled binding partner was diluted in serial dilution, starting with 752 µM of BSA and 1.6 µM of SIRT2 in a total volume of 50 µL. The fluorescently labeled peptide was added with a volume of 50 µL to the diluted binding partner with a final concentration of 50 nM of BSA and 10 nM of SIRT2. The solution was transferred to the 384-well plate, and the fluorescence intensity readout was performed with an Envision 2104 Multilabel plate reader with λ_Ex_ = 485 ± 14 nm and λ_Em_ = 535 ± 40 nm (one S-polarized and one P-polarized). The anisotropy (A) was calculated with the following equation:

$$A_{\mathrm{obs}}=\frac{{\mathrm{Fi}}_{S}-{\mathrm{Fi}}_{P}}{{\mathrm{Fi}}_{S}+2\cdot {\mathrm{Fi}}_{P}}$$

where Fi_S_ is the measured fluorescence intensity with the parallel filter and Fi_P_ is the measured fluorescence intensity with the perpendicular filter. The bound fraction [FB] was calculated from the resulting anisotropy (A_obs_) using the following equation:

$$\left[\mathrm{FB}\right]=\frac{A_{\mathrm{obs}}-A_{f}}{\left(A_{b}-A_{\mathrm{obs}}\right)\cdot Q+A_{\mathrm{obs}}-A_{f}}$$

where A_f_ represents the anisotropy of free **F4** without a binding partner, A_b_ the anisotropy of **F4** in the complex with the binding partner at saturation, and Q is the ratio of fluorescence intensities of bound **F4** versus free **F4**, which means that Q is < 1 when fluorescence is quenched upon binding. The total fluorescence intensities (Fi) were calculated with Fi = Fi(P-channel) + 2∗Fi(S-channel). [FB] was plotted as a function of the binding partner concentration, and the K_D_ value was determined with a nonlinear curve fit of the quadratic binding equation:

$$\left[\mathrm{FB}\right]=\frac{\left(K_{D}+L_{T}+R_{T}\right)-\sqrt{{\left(K_{D}+L_{T}+R_{T}\right)}^{2}-4R_{T}L_{T}}}{L_{T}}$$

where R_T_ is the total concentration of the unlabeled binding partner, L_T_ is the total concentration of the labeled peptide **F4**, and K_D_ is the dissociation constant. For a better overview, the binding curves are shown in a semi-logarithmic plot where the data were fitted to the following equation. This equation was also used to determine the K_D_ value of the sirtuin binding curves.

$$\left[\mathrm{FB}\right]=Y_{\min}+\frac{Y_{\max}-Y_{\min}}{1+10^{(\log K_{D}-R)\cdot n}}$$

### 4.12. Determination of Z′ Value and S/N Ratio

The Z′ factor was determined using a full 96-well plate (or 96 wells in a 384-well plate and 96 wells in a 1536-well plate), and half the plate contained a reaction mixture with 1 µM of **F4**, 500 µM of NAD^+^, and 10 nM of SIRT2 representing 100%, and the other wells contained the same reaction mixture without SIRT2 and represented the 0% value. Fluorescence readouts were performed with the settings described above after approximately 90% product formation (1 h). The Z′ factor was determined with the following equation, where SD indicates the standard deviation of the 100% values and the 0% values.

$$\;Z^{\prime }=\frac{3{\mathrm{SD}}_{100\%}+3{\mathrm{SD}}_{0\%}}{\left|{\mathrm{mean}}_{100\%}-{\mathrm{mean}}_{0\%}\right|}$$

The signal to noise ratio (S/N) was determined using the values described above in the following equation:

$$S/N=\frac{{\mathrm{mean}}_{100\%}-{\mathrm{mean}}_{0\%}}{{\mathrm{SD}}_{0\%}}$$

## Figures and Tables

**Table 1:** Kinetic constants for SIRT2, SIRT3, and SIRT5 for selected substrates (Figure 2 and Figure 3). SD denotes the standard deviation of three independent replicates, and n.d. denotes not determined.

Enzyme	Substrate	K_M_ ± SD (nM)	k_cat_ ± SD × 10^3^ (s^−1^)	k_cat_/K_M_ (M^−1^s^−1^)
SIRT2	**F1**	16 ± 3	8.4 ± 1.9	536,000
	**F2**	39 ± 5	6.7 ± 1.0	175,000
	**F4**	33 ± 5	23 ± 3	697,000
	**F5**	43 ± 5	15 ± 2	335,400
	**Mcm1**	17 ± 2	6.8 ± 1.5	400,000
SIRT3	**F1**	>20 µM	>17	n.d.
	**F2**	>20 µM	>6	n.d.
	**F4**	530 ± 70	23 ± 6	44,000
	**F5**	990 ± 70	27 ± 6	27,000

**Table 2:** IC_50_ values for SIRT2 using different substrates and SIRT2 inhibitors. The structures of all substrates are shown in Figure S12, and the structures of the inhibitors used are shown in Figure S13. **Mcm1**_T denotes a buffer containing 0.1% Tween20 (*w*/*v*) instead of BSA. SD values denote the standard deviations of at least two independent replicates.

cmp			IC_50_ Value ± SD in µM or Inhibition in % at a Given Concentration						
[S]	K (µM)	[E] (nM)	NAM	S2iL5	cmp12	SMyr	KK-22	SirReal2	AGK2
**F4** (1 µM)	0.033	10	100 ± 5	0.34 ± 0.02	0.32 ± 0.04	0.015 ± 0.001	16 ± 1.2	0% @20 µM	0% @20 µM
**F4** (40 nM)	0.033	1	211 ± 7	0.034 ± 0.004	0.015 ± 0.002	0.00078 ± 0.00005	1.5 ± 1	25 ± 2% @20 µM	42 ± 4% @20 µM
**S2** (1 µM)	0.0053	10	340 ± 7	1.3 ± 0.3	0.93 ± 0.2	0.028 ± 0.005	30 ± 2	18 ± 2% @20 µM	0% @20 µM
**S2** (10 nM)	0.0053	0.5	600 ± 70	0.028 ± 0.015	0.019 ± 0.006	0.00058 ± 0.00013	2.1 ± 0.1	1.1 ± 0.1	32 ± 2% @20 µM
**Mcm1** (1 µM)	0.017	10	43 ± 6	2.4 ± 0.5	1.7 ± 0.2	0.093 ± 0.002	7 ± 3% @20 µM	0% @20 µM	0% @20 µM
**Mcm1_T** (1 µM)	0.017	10	45 ± 3	1.9 ± 0.1	14 ± 3	0.058 ± 0.025	0% @20 µM	0% @20 µM	0 % @20 µM
**S1** (1 µM)	0.15	10	120 ± 10	0.56 ± 0.02	0.41 ± 0.04	0.0099 ± 0.003	12 ± 0.5	5.5 ± 0.8	33 ± 5 % @20 µM
**C1** (20 µM)	-	100	12 ± 1	0.21 ± 0.01	0.048 ± 0.005	0.056 ± 0.011	0.41 ± 0.03	0.089 ± 0.009	12 ± 1
**C2** (1 µM)	-	100	42 ± 6	0.42 ± 0.02	0.24 ± 0.03	0.33 ± 0.01	24 ± 1%	22 ± 3% @20 µM	6 ± 6% @ 6 µM

## Data Availability

Not applicable.

## Supplementary Materials

The following supporting information can be downloaded at: https://www.mdpi.com/article/10.3390/ijms24087416/s1.
