# Supplementary material for: Continuous Fluorescent Sirtuin Activity Assay Based on Fatty Acylated Lysines
Source: Int J Mol Sci. 2023 Apr 18;24(8):7416. doi: 10.3390/ijms24087416 (PMC10138348; doi:10.3390/ijms24087416)
Supplement: Supplementary file 1 [file ijms-24-07416-s001.zip › ijms-2284334-supplementary.pdf]

Continuous Fluorescent Sirtuin Activity Assay Based on Fatty Acylated Lysines

*Matthes Zessin[1], Marat Meleshin[2], Sebastian Hilscher[1], Cordelia Schiene-Fischer[2], Cyril Barinka[3], Manfred Jung[4] and Mike Schutkowski[2]**

[1] Department of Medicinal Chemistry, Institute of Pharmacy, Martin-Luther-University Halle-Wittenberg, Halle/Saale (Germany)

[2] Department of Enzymology, Charles Tanford Protein Center, Institute of Biochemistry and Biotechnology, Martin-Luther-University Halle-Wittenberg, Halle/Saale (Germany)

[3] Institute of Biotechnology of the Czech Academy of Sciences, BIOCEV, Prumyslova 595, 252 50 Vestec (Czech Republic)

[4] Institute of Pharmaceutical Sciences, University of Freiburg, Albertstraße 25, 79104 Freiburg Germany

KEYWORDS

Histone deacetylases, sirtuins, fluorescence quenching, sirtuin inhibitors, myristoylated substrates, continuous activity assay, bovine serum albumin effect

Figure S1. UPLC-MS Spectra of compound F1. 3
Figure S2. UPLC-MS Spectra of compound F2. 4
Figure S3. UPLC-MS Spectra of compound F3. 4
Figure S4. UPLC-MS Spectra of compound F4. 5
Figure S5. UPLC-MS Spectra of compound Mcm1 5
Figure S6. UPLC-MS Spectra of compound Mcm2. 6
Figure S7. UPLC-MS Spectra of compound C2... 6
Figure S8. Fluorescence spectra of the different peptides with and without 30 µM BSA in the buffer. 7
Figure S9. Using different blocking agents in the buffer to determine the IC50.. 8
Figure S 10. Using different blocking agents in the buffer to determine the IC50. 9
Figure S11. Determination of the Z’ factor.. 10
Figure S12. Substrates used for the different activity assay to determine the IC50 values 11
Figure S13. SIRT2 inhibitors used in this study.. 12
Figure S14. Inhibition analysis of compound 12 from Vogelmann et al. 2022 with SIRT2. 13

Table S 1. Overview about the molecular weight of the synthesized compounds.

compound	calculated mass (g/mol)	m/z of $[M+2H]^{2+}$	found mass (g/mol)	calculated mass - found mass (g/mol)
F1	1544.83	773.34	1544.68	0,15
F2	1572.88	787.25	1572.5	0.38
F3	1584.89	793.55	1585.1	-0.21
F4	1612.95	807.35	1612.7	0.25
Mcm1	1299.58	650.71	1299.42	0.16
Mcm2	1339.64	670.71	1339.42	0.22
C2	977.27	489.46	976.92	0.35

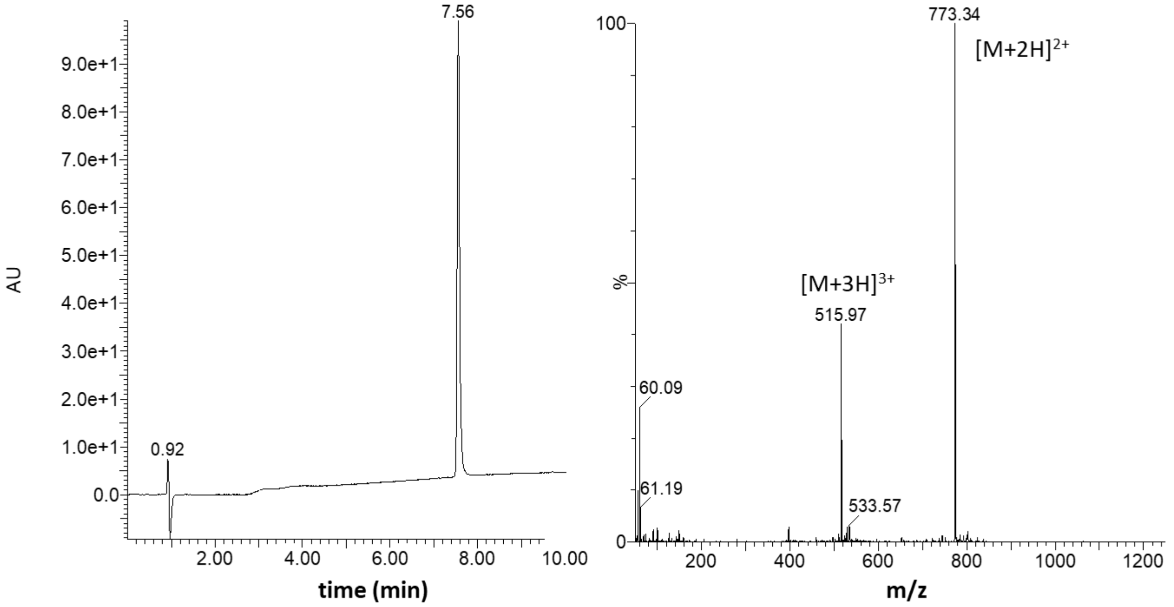

Figure S1. UPLC-MS Spectra of compound **F1**. **A.** The photometric analysis was done between 200 to 400 nm. **B.** Mass spectra of the peak at 7.56 min.

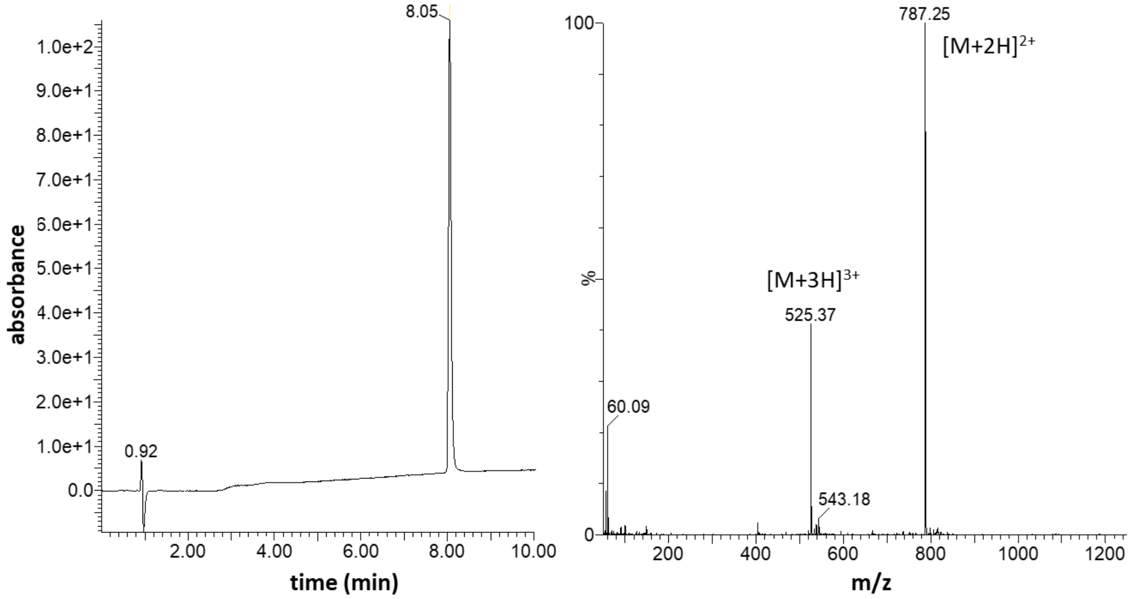

Figure S2. UPLC-MS Spectra of compound **F2**. **A.** The photometric analysis was done between 200 to 400 nm. **B.** Mass spectra of the peak at 8.05 min.

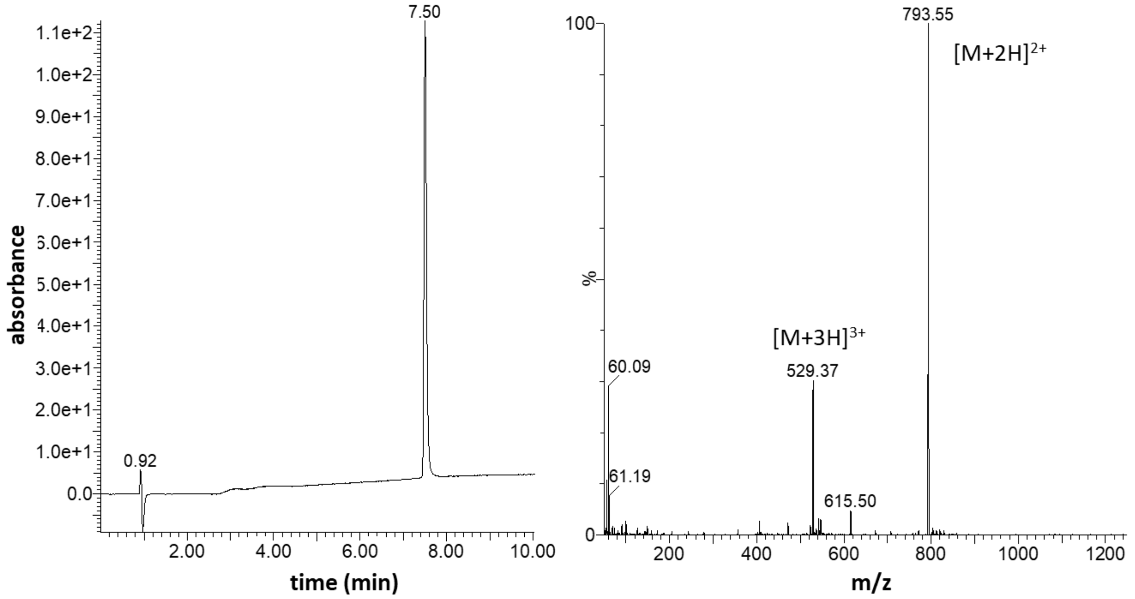

Figure S3. UPLC-MS Spectra of compound **F3**. **A.** The photometric analysis was done between 200 to 400 nm. **B.** Mass spectra of the peak at 7.50 min.

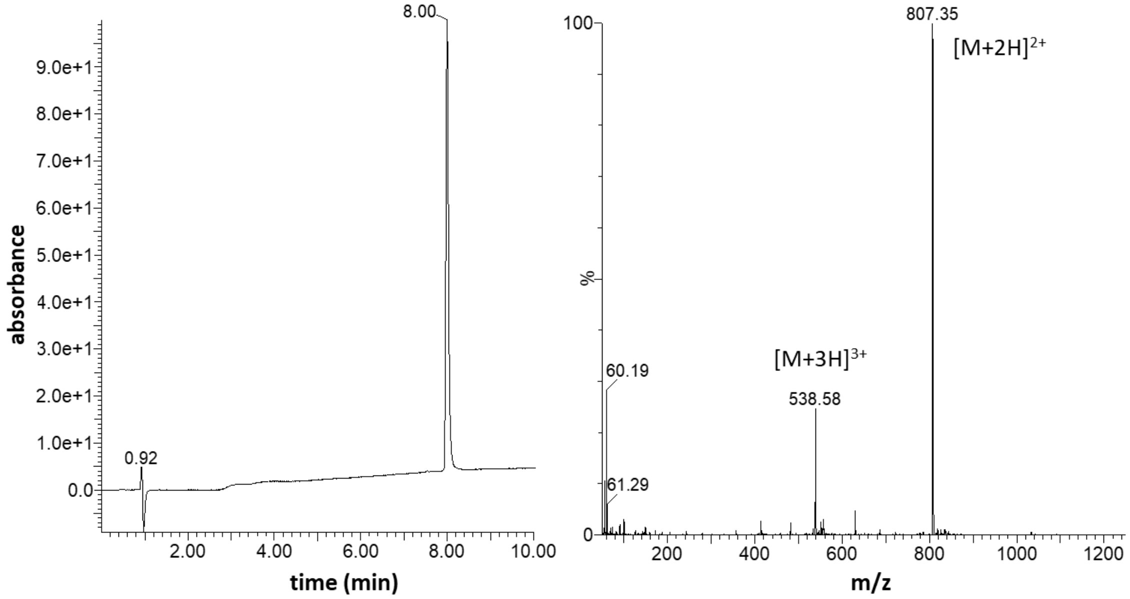

Figure S4. UPLC-MS Spectra of compound **F4**. **A.** The photometric analysis was done between 200 to 400 nm. **B.** Mass spectra of the peak at 8.00 min.

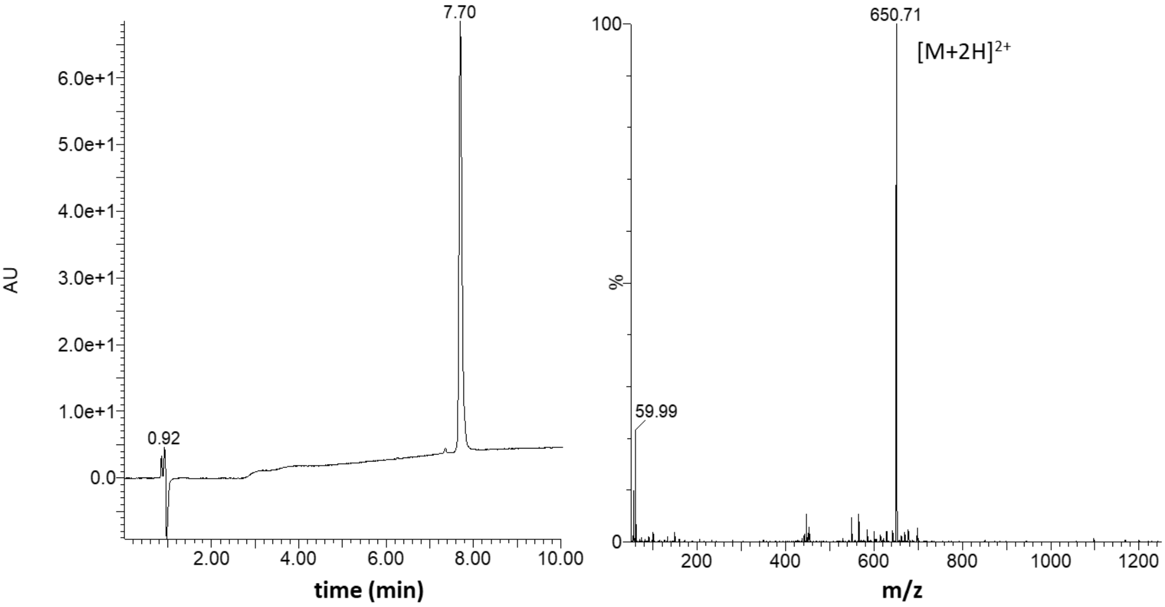

Figure S5. UPLC-MS Spectra of compound **Mcm1**. **A.** The photometric analysis was done between 200 to 400 nm. **B.** Mass spectra of the peak at 7.70 min.

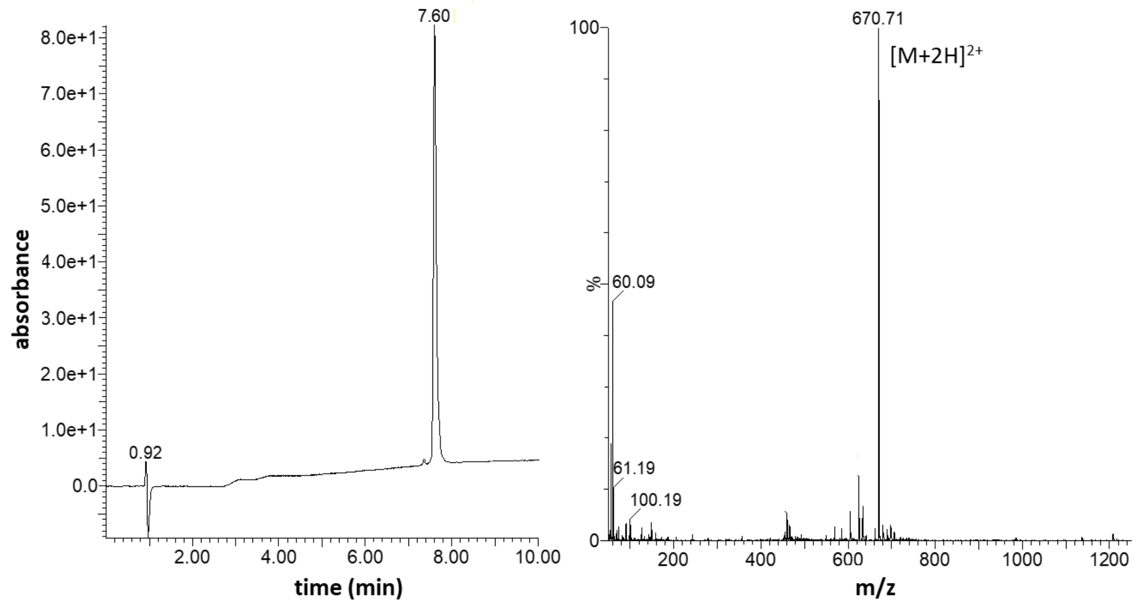

Figure S6. UPLC-MS Spectra of compound **Mcm2**. **A.** The photometric analysis was done between 200 to 400 nm. **B.** Mass spectra of the peak at 7.60 min.

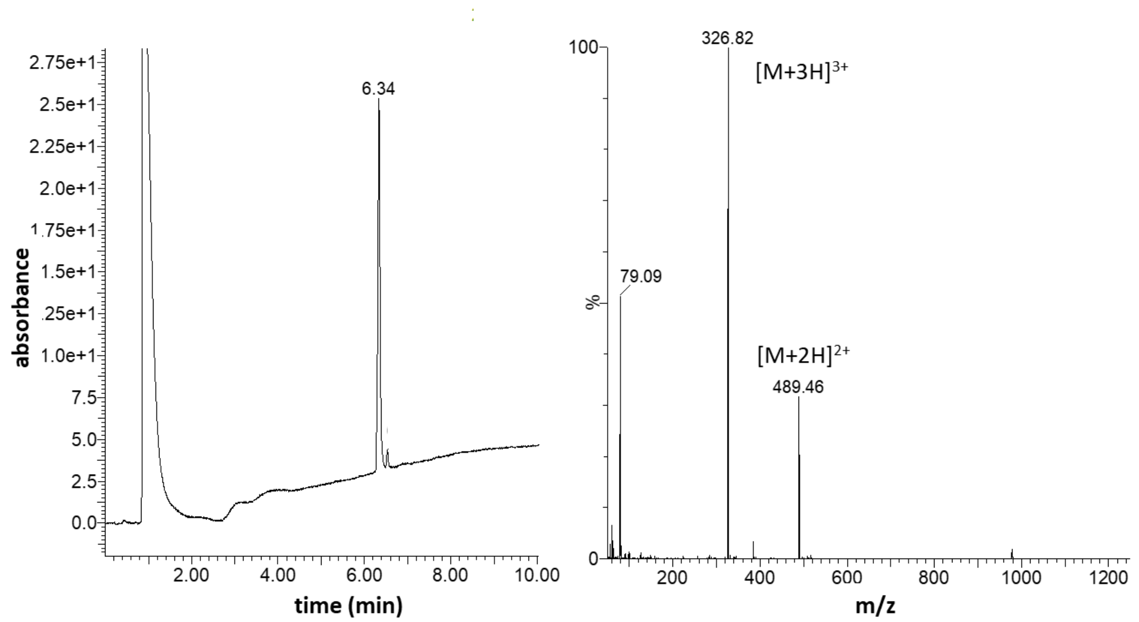

Figure S7. UPLC-MS Spectra of compound **C2**. **A.** The photometric analysis was done between 200 to 400 nm. **B.** Mass spectra of the peak at 6.34 min.

	Quenching efficiency Q_E (%)	
compound	without 30 µM BSA	with 30 µM BSA
Mcm1	60.2	58.4
Mcm2	18.5	49.4
F1	7.9	58
F2	14.1	51
F4	0.9	65
F5	4.9	50

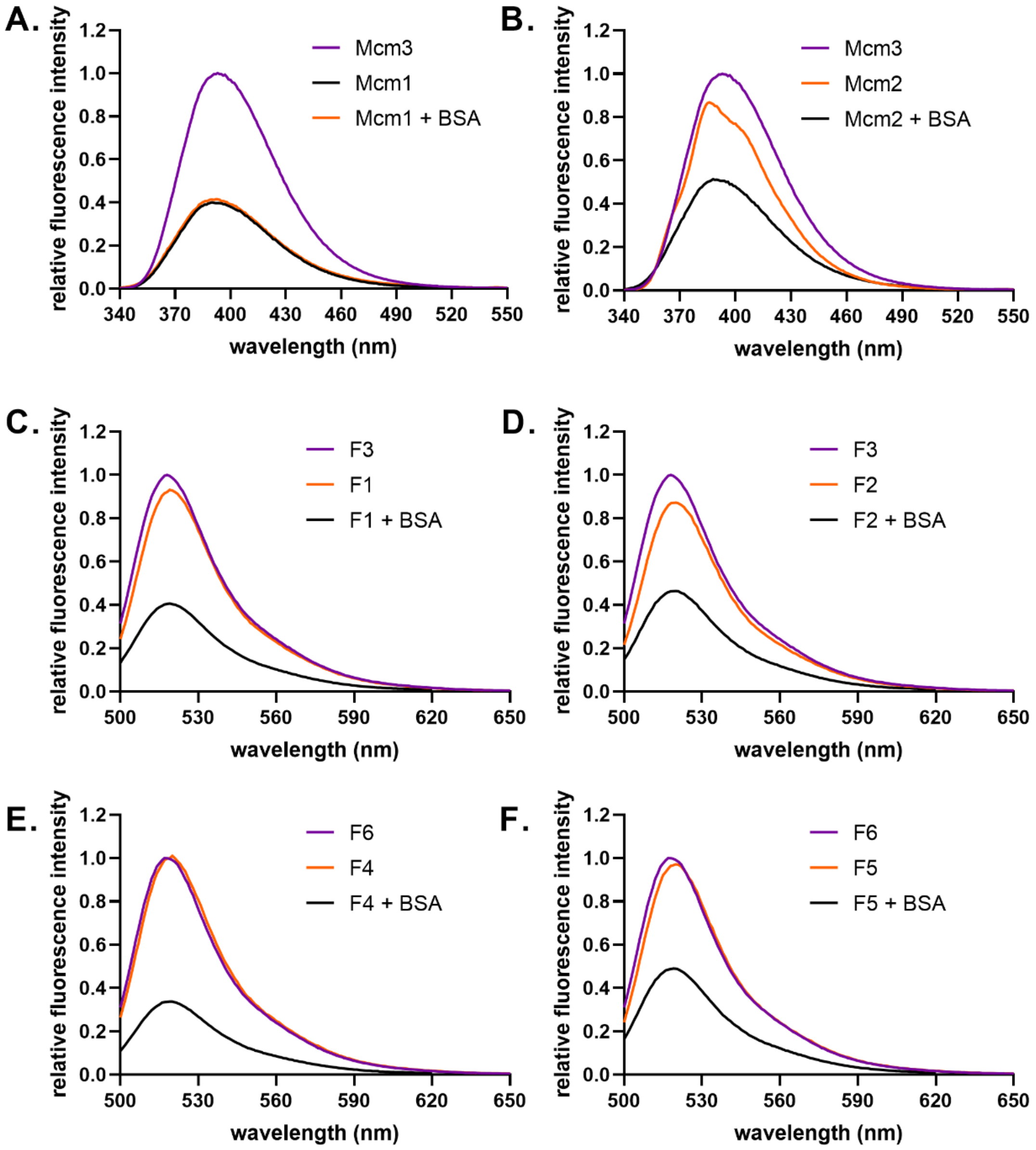

Figure S8. Fluorescence spectra of the different peptides with and without 30 µM BSA in the buffer. Spectra were recorded with excitation wavelengths at the absorbance maximum of the appropriate compound at a substrate concentration of 1 µM.

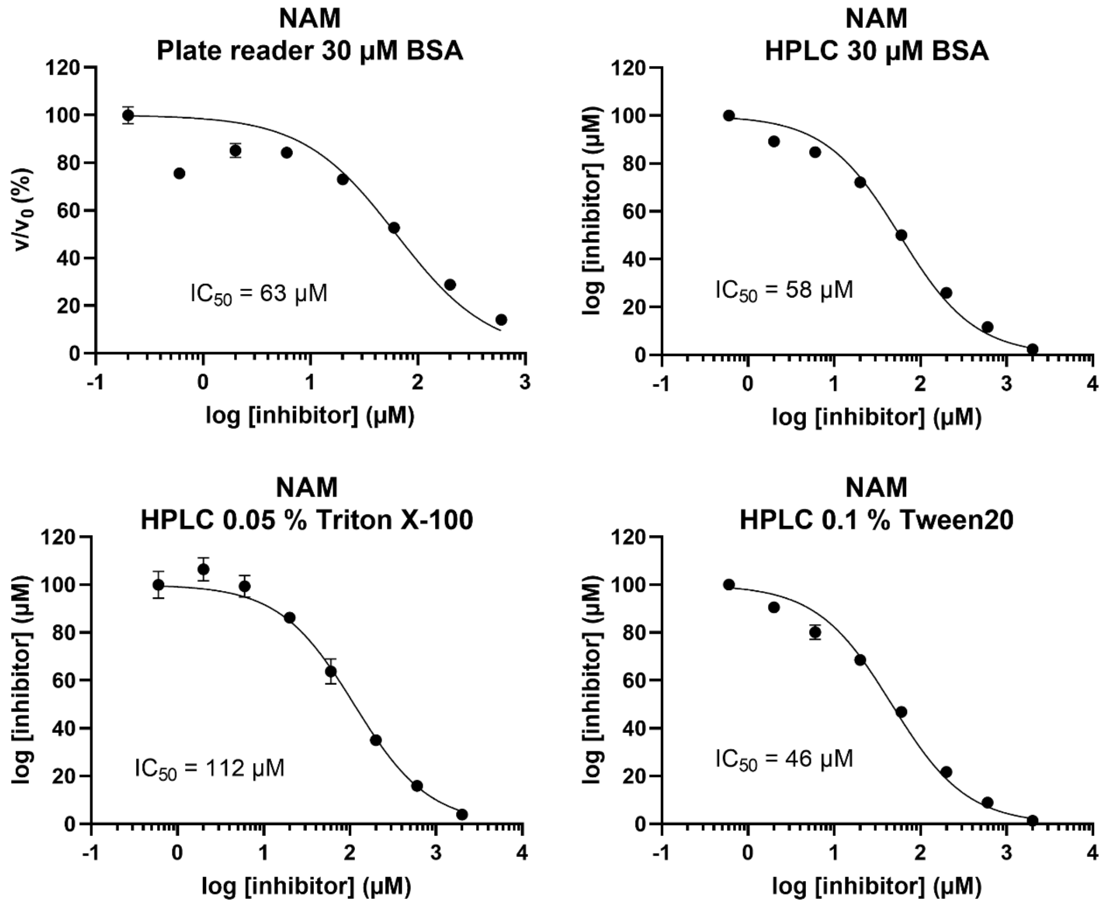

Figure S9. Using different blocking agents in the buffer to determine the IC50 value of SIRT2 for the noncompetitive inhibitor (against deacylation reaction) nicotinamide using compound F4 as a substrate. The IC50-value calculation was done as described in the method part. For HPLC measurements the substrate concentration was 1 µM and the SIRT2 concentration was 10 nM. Peptide substrate, NAD^+ and inhibitor was incubated at 25 °C and the reaction was started with enzyme addition. After 10 to 15 min (dependent on the enzyme activity) the reaction was quenched by adding 1 % TFA in water (v/v) and product formation was monitored using analytical HPLC at a detection wavelength of 450 nm. The reaction buffer was the Sirtuin assay buffer supplemented with 30 µM BSA top or 0.05 % Triton X-100 or 0.1 % Tween20.

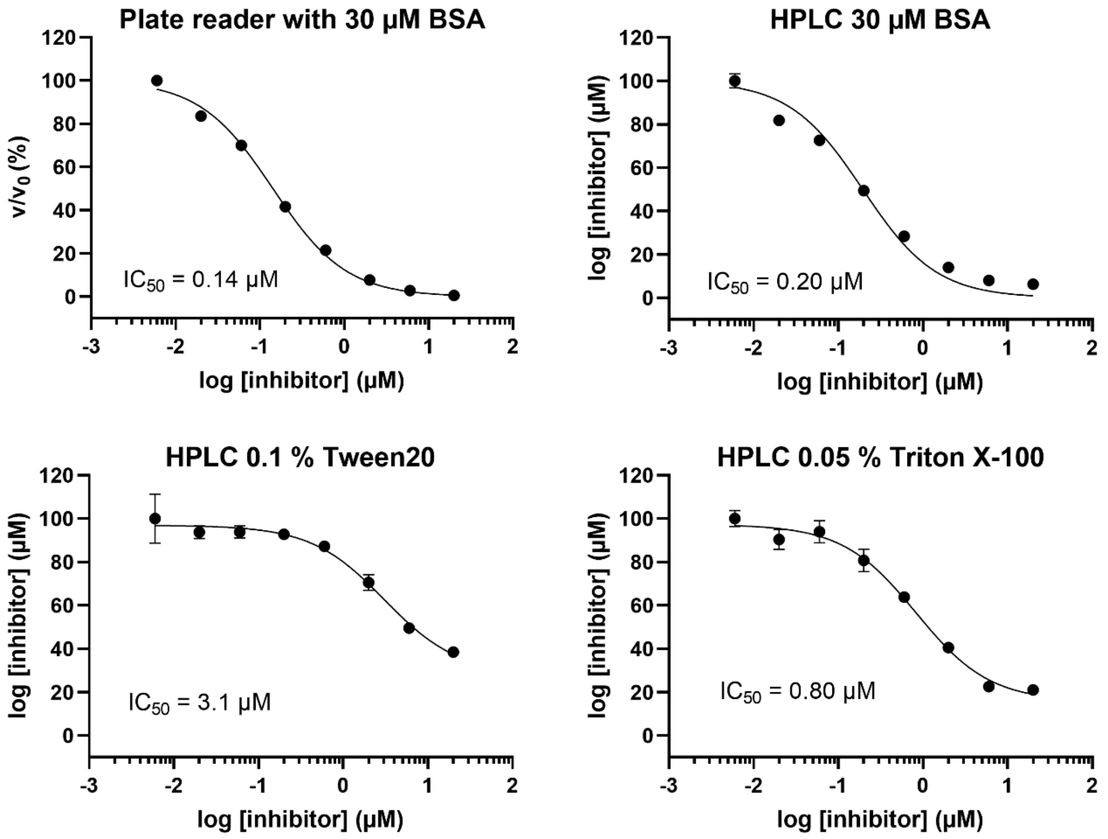

Figure S 10. Using different blocking agents in the buffer to determine the IC50 value of SIRT2 and a competitive SIRT2 inhibitor (for the diacylation reaction) and F4 as the substrate. The IC50-value calculation was done as described in the method part. For HPLC measurements the substrate concentration was 1 µM and the SIRT2 concentration was 10 nM. Peptide substrate, NAD^+ and inhibitor was incubated at 25 °C and the reaction was started with enzyme addition. After 10 to 15 min (dependent on the enzyme activity) the reaction was quenched by adding 1 % TFA in water (v/v) and product formation was monitored using analytical HPLC at a detection wavelength of 450 nm.

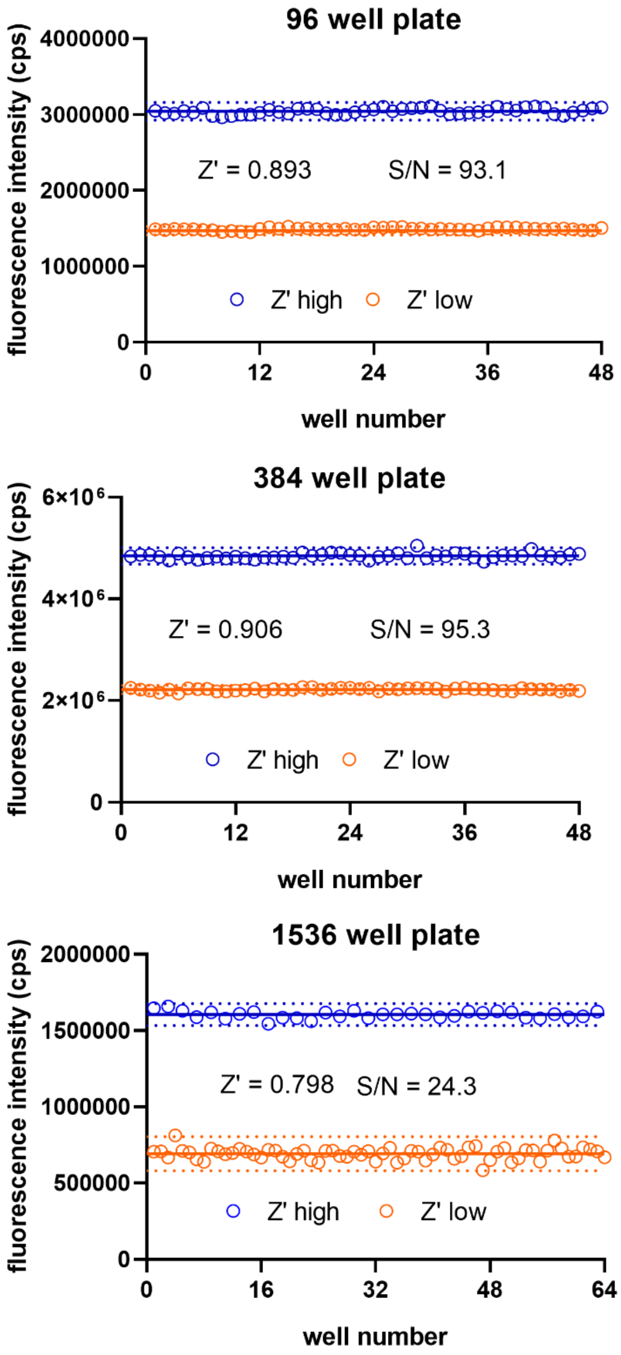

Figure S11. Determination of the Z' factor. The substrate F4 and NAD$^+$ were incubated in a black 96- or 384 well plate with a final concentration of 1 µM and 500 µM. The reaction was started with the addition of SIRT2 at a concentration of 10 nM for 48 wells for the Z' high sample and with buffer for the 48 wells for the Z' low sample. The fluorescence readout was done continuously every 30 s with λ_{Ex} 485 ± 14 nm and λ_{Em} 535 ± 25 nm. Fluorescence intensity values were taken for one time point after 90 % product formation for the Z' factor and the S/N ratio calculation. For the 1536 well plate analysis the reaction was started in a 96 well transparent well plate with V bottom and transferred to the black 1536 well plate and the readout was started.

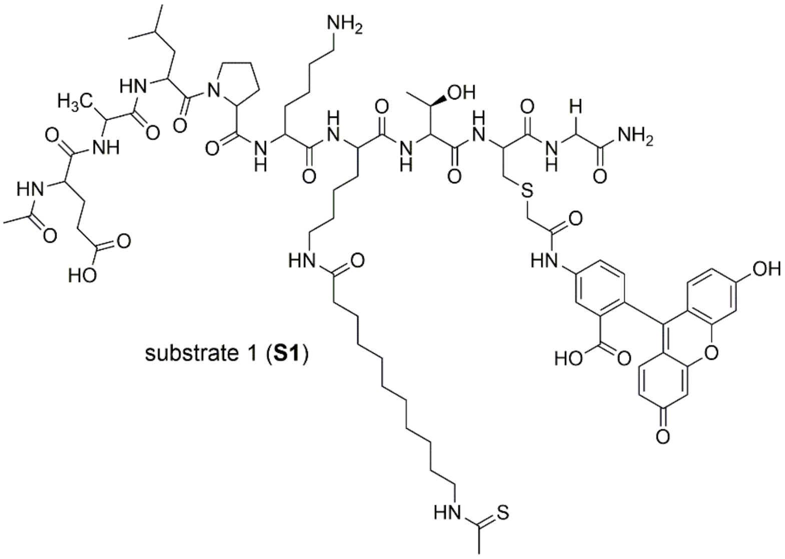

substrate 1 (**S1**)

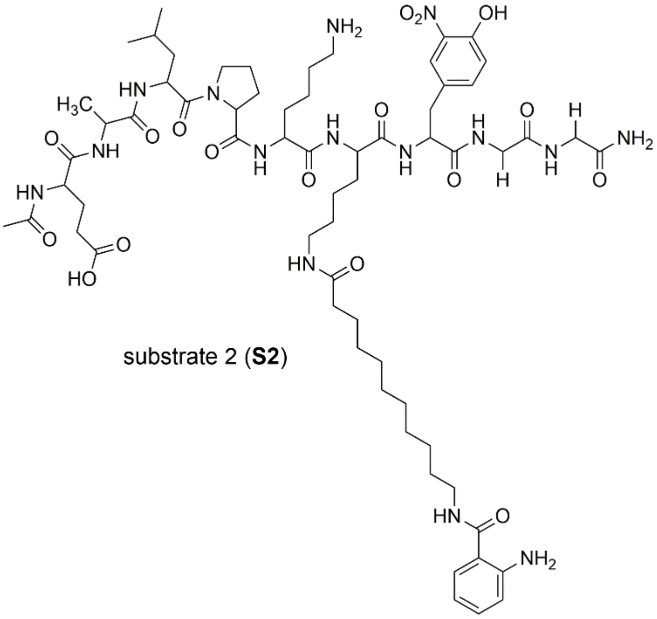

substrate 2 (**S2**)

Figure S12. Substrates used for the different activity assay to determine the IC50 values of different inhibitors.

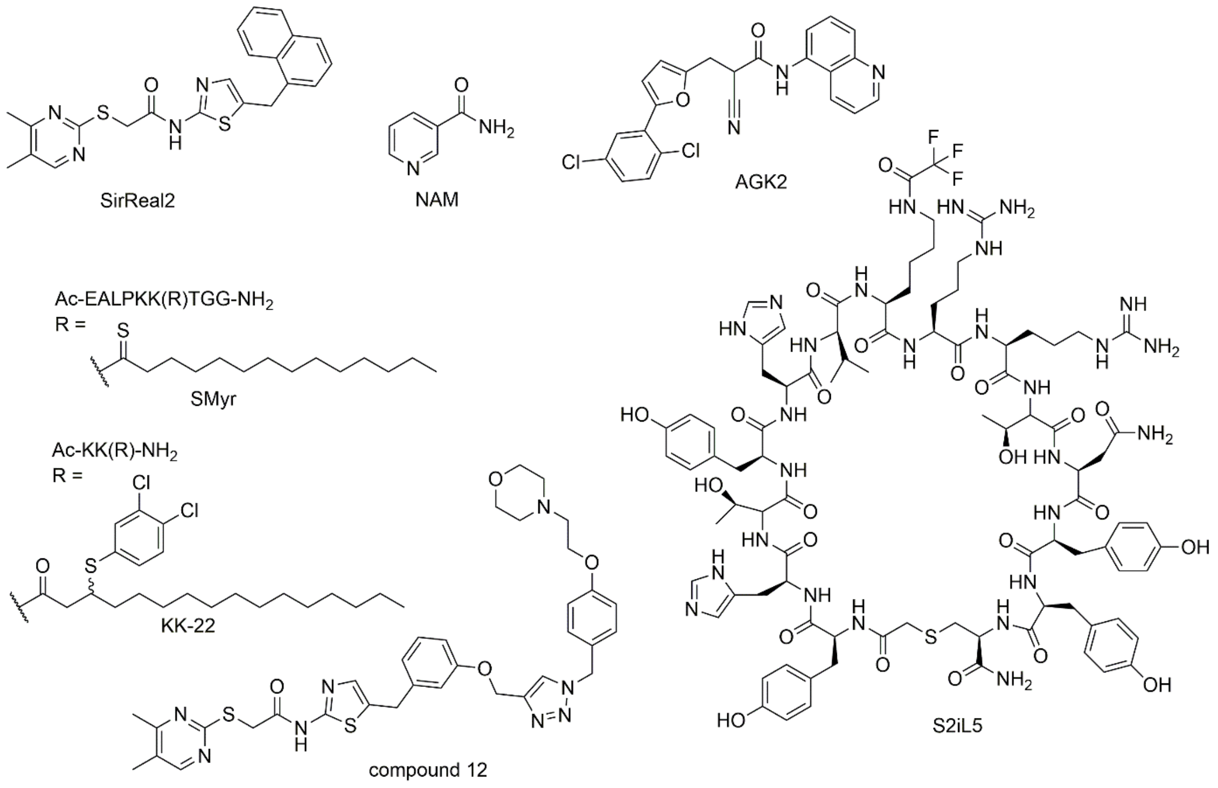

Figure S13. SIRT2 inhibitors used in this study.

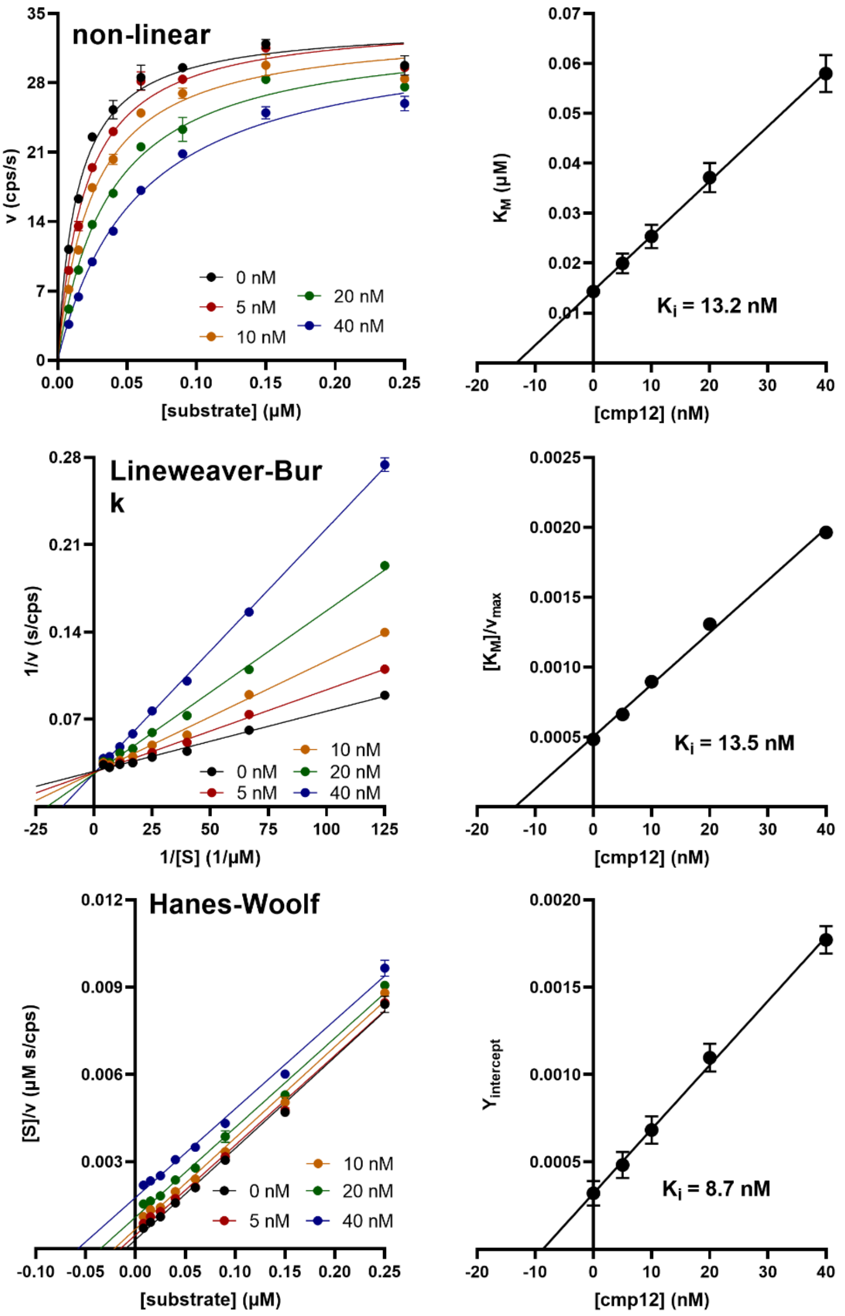

Figure S14. Inhibition analysis of compound 12 from Vogelmann et al. 2022 with 1 nM SIRT2, 500 µM NAD+ and different concentrations of F4 and different concentration of cmp 12. Structure of compound 12 is shown in Fig S12. Inhibition type analysis suggest that compound 12 is a competitive SIRT2 inhibitor.
